# Signatures of illness in children requiring unplanned intubation in the pediatric intensive care unit: A retrospective cohort machine-learning study

**DOI:** 10.3389/fped.2022.1016269

**Published:** 2022-10-19

**Authors:** Michael C. Spaeder, J. Randall Moorman, Liza P. Moorman, Michelle A. Adu-Darko, Jessica Keim-Malpass, Douglas E. Lake, Matthew T. Clark

**Affiliations:** ^1^ Department of Pediatrics, Division of Pediatric Critical Care, School of Medicine, University of Virginia, Charlottesville, VA, United States; ^2^ Center for Advanced Medical Analytics, School of Medicine, University of Virginia, Charlottesville, VA, United States; ^3^ Department of Medicine, Division of Cardiovascular Medicine, School of Medicine, University of Virginia, Charlottesville, VA, United States; ^4^ Nihon Kohden Digital Health Solutions, Irvine, CA, United States; ^5^ Department of Acute and Specialty Care, School of Nursing, University of Virginia, Charlottesville, VA, United States

**Keywords:** respiratory failure, intensive care units, pediatric, intubation, machine learning, child, infant

## Abstract

Acute respiratory failure requiring the initiation of invasive mechanical ventilation remains commonplace in the pediatric intensive care unit (PICU). Early recognition of patients at risk for respiratory failure may provide clinicians with the opportunity to intervene and potentially improve outcomes. Through the development of a random forest model to identify patients at risk for requiring unplanned intubation, we tested the hypothesis that subtle signatures of illness are present in physiological and biochemical time series of PICU patients in the early stages of respiratory decompensation. We included 116 unplanned intubation events as recorded in the National Emergency Airway Registry for Children in 92 PICU admissions over a 29-month period at our institution. We observed that children have a physiologic signature of illness preceding unplanned intubation in the PICU. Generally, it comprises younger age, and abnormalities in electrolyte, hematologic and vital sign parameters. Additionally, given the heterogeneity of the PICU patient population, we found differences in the presentation among the major patient groups – medical, cardiac surgical, and non-cardiac surgical. At four hours prior to the event, our random forest model demonstrated an area under the receiver operating characteristic curve of 0.766 (0.738 for medical, 0.755 for cardiac surgical, and 0.797 for non-cardiac surgical patients). The multivariable statistical models that captured the physiological and biochemical dynamics leading up to the event of urgent unplanned intubation in a PICU can be repurposed for bedside risk prediction.

## Introduction

Acute respiratory failure requiring the initiation of invasive mechanical ventilation remains commonplace in the pediatric intensive care unit (PICU) (1). Emergent endotracheal intubation in children is associated with an increased risk of complications as compared to intubations performed electively (2). Early recognition of patients at risk for respiratory failure may provide clinicians with the opportunity to intervene and potentially improve outcomes (3).

Predictive analytics leverages physiologic and biochemical data in the development of algorithms that can identify signatures of illness present early in the process of clinical decompensation (4–6). Display of algorithmic-derived scores or indices alert care providers to patients at risk for clinical decompensation. Our group previously found a signature of respiratory distress leading to unplanned intubation in the adult intensive care unit (ICU) population that we validated externally at another site (3, 7, 8). Display of a risk estimate for this event in a surgical ICU was associated with a fall in septic shock by 50% that was not matched in a medical ICU that did not have the display (9). Through the development of a random forest model to identify patients at risk for requiring urgent unplanned intubation, we tested the hypothesis that subtle signatures of illness are present in physiological and biochemical time series of PICU patients in the early stages of respiratory decompensation (10).

## Materials and methods

### Study design and definitions

We performed a retrospective cohort study from January 2014 to May 2016 at the University of Virginia (UVA) Children’s Hospital. We included all admissions to the PICU, a 17-bed combined cardiac and medical/surgical unit with stored continuous physiologic monitoring data. We defined admission as a unique hospitalization that included PICU stay irrespective of the number of transitions into and out of the PICU. The Institutional Review Board at the University of Virginia approved this study. We followed the Strengthening the Reporting of Observational Studies in Epidemiology guidelines (11).

We identified all intubations performed in the UVA Children's Hospital associated with a PICU admission during the period of study through querying of individual electronic health records. We established our primary end point of an unplanned intubation event based on the time of the event recorded in the electronic health record and a non-elective indication for intubation as documented in the UVA contribution to the National Emergency Airway Registry for Children (NEAR4KIDS) database. We also performed a sensitivity analysis using only the computable phenotype of intubation event recorded in the electronic health record without regard to patient location or indication for intubation (includes e.g., patients intubated in Operating Room by Anesthesia prior to surgery) to evaluate the importance of manual chart review.

The 12-hour window prior to each unplanned intubation event was identified and classified (provided the patient was in the PICU and not already intubated) as case data. We classified data collected at all other times in patients with or without unplanned intubation events as control data. We excluded intubations within 15 min of PICU admission, or those within one hour of a prior extubation. We excluded admissions without archived physiologic monitoring data due to technical complications. Multiple unplanned intubation events in individual patients were treated as independent events for the purposes of analysis as each 12-hour window of case data was distinct.

We excluded times during which a patient was mechanically ventilated. Presence of mechanical ventilation was determined by extracting the ventilator respiratory rate flowsheet vital sign from the electronic data warehouse. We defined mechanical ventilation as starting at the time of the first ventilator respiratory rate and ending at the time of the last ventilator respiratory rate. The period of mechanical ventilation was split when the time between consecutive measurements from a patient was longer than 12 h, and identified the patient as not ventilated in the interim (see Supplementary Figure A1).

### Physiologic data acquisition and predictors

Continuous cardiorespiratory monitoring consisted of waveforms (3 leads of ECG sampled at 240 Hz, pulse plethysmography at 120 Hz, and invasive blood pressure tracings at 120 Hz) and vital signs (heart rate, respiratory rate, peripheral oxygen saturation, invasive blood pressure, ventilator measured respiratory rate, and sample-and-hold non-invasive blood pressure) sampled at 0.5 Hz. The GE monitor (GE Healthcare, Chicago, IL) reported sample-and-hold non-invasive blood pressure at 0.5 Hz, while monitor-smoothed invasive blood pressure was measured continuously. We calculated, in 30-minute windows with 50% overlap, the following 18 measures: the mean and standard deviation of heart rate (HR), respiratory rate (RR), pulse oximetry (SO2); mean non-invasive systolic and diastolic blood pressure, or invasive blood pressure in its absence (SBP and DBP); the three pairwise cross-correlations between HR, RR, and SO2; standard deviation of heart inter-beat intervals (sRRI); local dynamics score (LDs) and local dynamics density (LDd) of heart inter-beat intervals (12); coefficient of sample entropy (COSEn) (13); the slope of log variance vs. log scale between scales 4 and 12 for detrended fluctuation analysis of heart inter-beat intervals (DFA) (14); the probability of atrial fibrillation (15). Non-invasive blood pressure was cycled more frequently than every 30-minutes in 95% of epochs in which it was available. Low quality EKG data were excluded from cardiac dynamic calculations (16, 17). LDs and LDd quantify how many heart inter-beat intervals match very many or no other intervals. COSEn quantifies the repeatability of the heart inter-beat interval time series. Finally, DFA quantifies the way in which the variability of heart inter-beat intervals depends upon time scale. The cardiorespiratory dynamics measured from continuous cardiorespiratory monitoring were calculated as described in (18) using CoMET® (AMP3D Inc., a Nihon Kohden Company, Charlottesville, VA). Cardiorespiratory dynamics were not available to the care team.

Vital signs (heart rate, respiratory rate, oxygen saturation, blood pressure, and temperature) were extracted from flowsheets. Additionally, we extracted fraction of inspired oxygen and supplemental oxygen flowrate and combined it with peripheral oxygen saturation to estimate the PO_2_ to FiO_2_ ratio (ePFR) following the methods of Gadrey, et al*.* (19, 20). Eleven frequently measured laboratory measurements (serum sodium, potassium, chloride, bicarbonate, blood urea nitrogen (BUN), creatinine, glucose, calcium, white blood cell count, hematocrit, platelet count) were extracted from the electronic data warehouse. Anion gap and BUN to creatinine ratio were included as features. We combined these intermittent features with continuously measured features using sample-and-hold. Vital signs and labs older than 24 and 48 h, respectively, were censored. Finally, we included age as a continuous predictor.

We also performed a sensitivity analysis by excluding features from continuous cardiorespiratory monitoring, both for the chart reviewed unplanned intubations and the computable phenotype.

### Subgroup analysis

The study cohort includes patients admitted for a variety of reasons. To investigate differences in physiological signatures between these populations, we identified the patient type for each admission in our retrospective analysis. We defined patient type for each admission based on operating room records: admissions without any operating room records were identified as medical patients, those with a thoracic cardiovascular operating room record were identified as cardiac surgery, and those with any other type of operating room record were identified as non-cardiac surgery.

### Physiological signature of illness

We construct physiological signatures of illness specific to age and each patient type in the cohort. For this, predictiveness curves were calculated to display the independent association of vital signs, laboratory values, and continuous cardiorespiratory monitoring parameters with unplanned intubation. To reduce bias due to repeated measures and missing data, we used a bootstrapping technique to estimate the predictiveness curves. For each patient type and for all patients, we randomly sampled eight measurements within 12 h before unplanned intubation and eight measurements from each admission without an intubation event or far from the time of intubation. We calculated the relative risk of unplanned intubation at each decile of the sampled variable and each decile of age over the surrounding quintile. We repeated this process of sampling and calculating relative risk 30 times then averaged to obtain a bootstrapped predictiveness curve and displayed the results as a heat map. Toddenroth and colleagues present an excellent introduction to the construction and interpretation of heat maps in clinical research (21).

### Model development

We developed a model on the entire cohort and used cross validation for estimating performance characteristics. Modeling was performed in R (R Foundation for Statistical Computing, Vienna, Austria) using the *randomForest* packages (22, 23). We used random forest to construct a multivariable model to account for the high-dimensional relationships between predictors, and especially to account for the known age-dependence of many features. We justify its use over other logistic regression based on our prior finding that random forest more accurately captured the influence of age in the signature of sepsis in this population (24). As predictors, we used 18 measures from continuous cardiorespiratory monitoring, 8 vital signs, 13 laboratory measurements, and age. We performed a sensitivity analysis by including a random variable to identify features that were not significantly associated with unplanned intubation. We imputed missing data based on patient age. We first identified the deciles of age and calculated the median of each feature in each decile. Missing values were then imputed with the median for the decile associated with the age of the patient at the missing measurement time.

We constructed a random forest model (25) to identify data for patients that had an unplanned intubation event in the next 12 h. The forest consisted of 800 classification trees, and 6 features (the square root of the number of features) were sampled as candidates at each split (26). We adjusted for the imbalance between the intubation event and non-intubation event data by bootstrapping the event data and sampling the same number of non-event data. The output of the model was the fraction of trees that classified a record as an event. We divided this output by the average value to obtain predicted relative risk, then multiplied by the average probability of unplanned intubation in the next 12 h to get predicted probability.

The predicted risk was calculated using 5-fold cross validation (27). Briefly, hospital admissions were randomly sampled into 5 groups containing approximately 20% of admissions. We used the first fold (20%) as the test set, and we built a model on the remaining 4 folds (80% of admissions). The predicted risk for the test set was estimated using this model, and the procedure repeated for each of 5 folds admissions. This patient-wise method provides less biased performance estimates than cross-validation that selects folds based on row, because in the latter method data from an individual admission may be included in many folds and therefore be used for training and testing.

We also followed this model development and validation procedure to compare performance characteristics for models to predict: the computable phenotype for unplanned intubation excluding continuous cardiorespiratory monitoring features; the computable phenotype for unplanned intubation using all features; the chart reviewed events of unplanned intubation excluding continuous cardiorespiratory monitoring features.

### Performance characteristics of predictive models

The model developed here is intended for continuous risk estimation. We contextualized the performance and potential clinical impact of our predictive models relative to the HRC index. We evaluated our model by plotting the time-course of cross-validated predicted risk leading up to the time of unplanned intubation event. Early detection requires not only high-predicted risk before diagnosis, but also an increasing risk to indicate worsening patient status towards critical illness. We also calculated the area under the receiver operating characteristic (AUC) based on cross-validated predictions. The AUC is equivalent to the C-statistic: it is the probability that, when randomly drawing one predicted probability from case and control data, the case data has a higher prediction. A random classifier has the expected AUC of 0.5 and perfect discrimination has AUC of 1.0.

We find a surprisingly wide variety of methods for calculating AUC in the literature. One approach to achieve artificially high AUC is to report “encounter-based” AUCs, meaning that each patient is characterized by the highest AUC during the hospitalization. Thus, the score representing the case patient may have occurred long before or long after the diagnosis was made. This is not useful to the bedside clinician. It is not possible to compare two AUCs unless the data sets and the evaluation criteria are the same. Here we report AUC based on evaluation of all case and control records where a patient is at risk for unplanned intubation in the PICU.

## Results

Table 1 shows the study cohort. There were 2767 PICU admissions included in the study. When we require a non-elective indication for intubation using the UVA contribution to the NEAR4KIDS database we found 92 admissions with 116 unplanned intubations. The most common indications noted in the NEAR4KIDS database for unplanned intubations were respiratory failure (64%), both respiratory failure and hemodynamic instability (14%), and hemodynamic instability alone (12%). Patients who experienced unplanned intubation during their stay were younger and were more likely to have had cardiac surgery during their stay. When we did not require a non-elective indication from the UVA contribution to the NEAR4KIDS database we found 944 admissions using the computable phenotype alone.

**Table 1:** Demographics of the study population.

	No Event (*n* = 2675)	Event (*n* = 92)	*p*-value
Age, median (IQR)	3 yr (6 mo – 12 yr)	5 mo (1 mo – 3 yr)	*
Hospital LOS, median (IQR)	4 days (2 days – 10 days)	33 (18 days – 63 days)	*
In-hospital mortality, %	3.30%	18.50%	*
Male sex, %	54.20%	58.70%	0.39
Patient Type			
Medical, *n* (%)	1222 (45.7%)	19 (20.7%)	*
Non-cardiac surgery, *n* (%)	826 (30.9%)	26 (28.3%)	0.59
Cardiac surgery, *n* (%)	627 (23.4%)	47 (51.1%)	*

Abbreviations: IQR, interquartile range; LOS, length of stay.

* indicates *p* < 0.0001 based on Wilcoxon rank sum testing (numerical data) or standard error for the difference of proportions.

**Figure 1:**
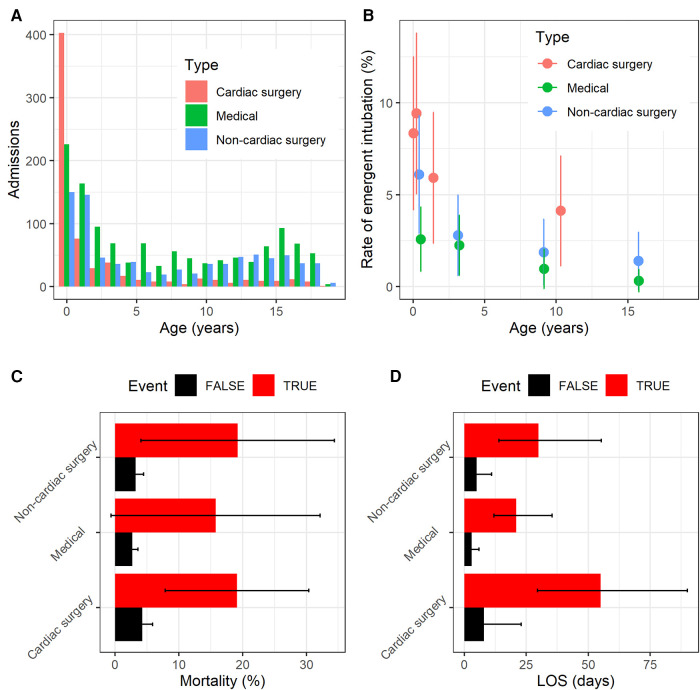
Characteristics of the study population. (**A**) Histogram of admissions for each patient type as a function of age. (**B**) Rate of unplanned intubation for each patient type as a function of age. (**C**) Mortality rate for each patient type with (red) and without (black) unplanned intubation. Error bars are 95% confidence interval. (**D**) Median length of stay in days for each patient type with (red) and without (black) unplanned intubation. Error bars show the IQR.

Figure 1 shows the characteristics of the study cohort. In Figure 1A, patients admitted for cardiac surgery were predominantly infants admitted with complex congenital heart disease, while medical and non-cardiac surgery patients were more distributed across the pediatric age range. In Figures 1B–D, the estimates are made at each quartile of age for the specific patient type. Unplanned intubation was more likely for younger patients regardless of patient type, see Figure 1B. Unplanned intubation increased length of stay all patient types and ages, and increased mortality for most in Figures 1C,D.

**Figure 2:**
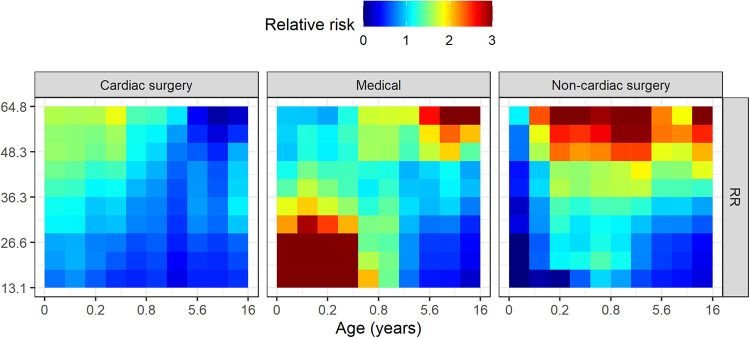
Empirical relative risk for cardiac surgery (left), medical (center), and non-cardiac surgery (right) patients as a function of age and mean respiratory rate from continuous monitoring data. On each panel, younger patients are to the left, and slower respiratory rates are to the bottom. Each colored tile estimates the relative risk of unplanned intubation for each decile of age and respiratory rate based on the surrounding quintile. Deciles of respiratory rate and age remain the same across patient type panels. Higher relative risk is redder while lower is bluer.

**Figure 3:**
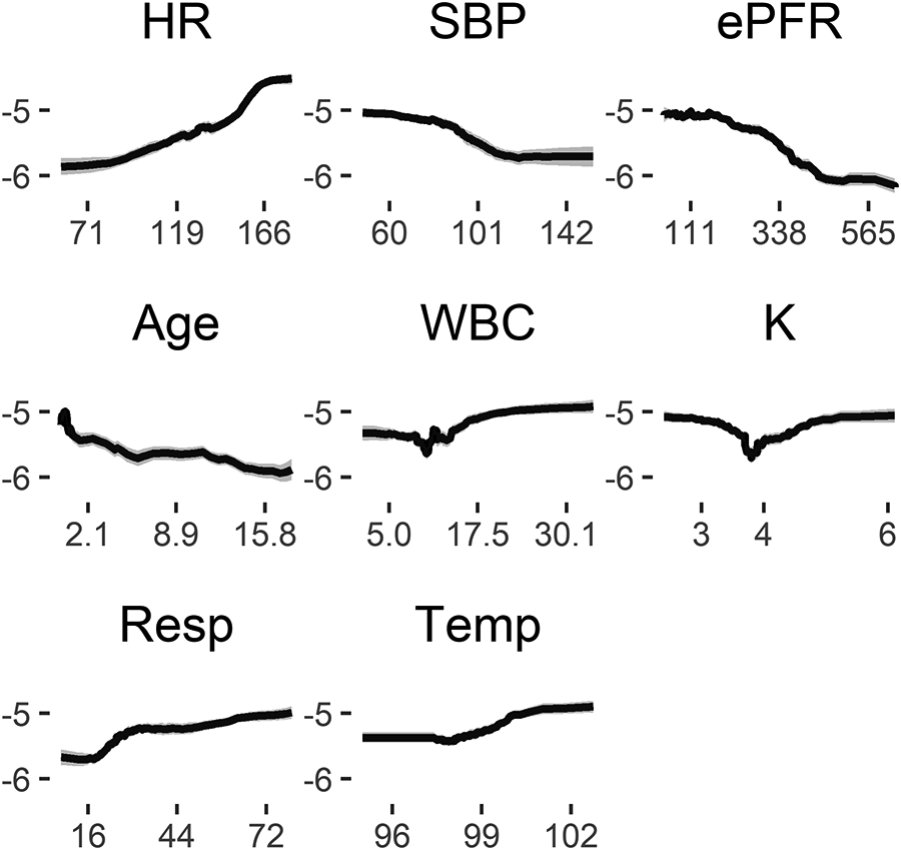
Risk profiles for exemplary features in the random forest model. These are marginal risk profiles that average out dependence on other features, such as the dependence of heart rate on age. The (natural) log odds of unplanned intubation in the next 12 h (ordinate) is shown as a function of the value of each measured variable (abscissa) holding all other features at their median values. Lower blood pressure, for example, is associated with increased risk for unplanned intubation independent of changes in any other feature.

Figure 2 shows an example of the physiological signature of unplanned intubation for cardiac surgery (left), medical (center), and non-cardiac surgery (right) patients. In each panel, each colored column estimates the empirical relative risk for unplanned intubation as a function of respiratory rate for one decile of age based on the surrounding quintile. That is, for all measurements in each decile of age and respiratory rate, we calculated the probability of unplanned intubation in the next 12 h and divided by the average rate of unplanned intubation in the cohort (0.0063). The youngest 10% of medical patients, for example, have about 3-fold more risk than average (redder) for unplanned intubation when respiratory rate is in the lower 30% (less than about 27 breaths per minute) independent of all other features. Averaging across all columns and panels yields an age and patient type marginalized risk profile for respiratory rate as shown in Figure 3. The deciles of respiratory rate and age are maintained across the rows and panels. There was a distinct physiological signature of illness for unplanned intubation depending on patient type. High respiratory rate is a signature of unplanned intubation for non-cardiac surgery patients and older medical patients, while in younger medical patients it is low respiratory rate and for cardiac surgery patients there is not a strong signature in respiratory rate. See Supplementary Figure A2 for all features.

Figure 3 shows the marginal risk profiles of several example features for the random forest model based on all patients. Each panel shows the log odds of unplanned intubation (ordinate) as a function of the value of each feature (abscissa). We estimated log odds by varying each feature across its range and estimated the log odds from the predicted probability while keeping all other features at their median values, then calculated the logit of predicted probability based on the model. Increasing heart rate from about 70 to 150 with all other features at the median values for the sample, for example, increases the log odds of unplanned intubation in the next 12 h from about −6 to −5, or an increase in probability from 0.0025 to 0.0067. Note that this marginalized representation removes the dependence captured by the model of each feature on age. See Supplementary Figure A3 for all features. All features were found to be more important than the random feature. Small changes in heart rate, respiratory rate, blood pressure, and others, alone or in combination, can be signatures of changing risk for unplanned intubation.

The AUC for the random forest model based on cross-validated predictions is 0.696 for all patients (0.692 for medical, 0.673 for cardiac surgery, and 0.727 for non-cardiac surgery). The AUC depends on the definition of case data (24): the AUC for the random forest model using a definition of 4 h before the event (rather than 12 h before) is 0.766 (0.738 for medical, 0.755 for cardiac surgical, and 0.797 for non-cardiac surgical). For comparison, the AUC of HRC monitoring for neonatal sepsis [that resulted in 20% mortality reduction (28)] within 24 h was 0.70 in the development cohort (29). In adults, prospective display of models for unplanned intubation and hemorrhage with AUC of 0.68 and 0.71, respectively (7), were associated with a 50% reduction of septic shock in an adult surgical ICU (9).

**Figure 4:**
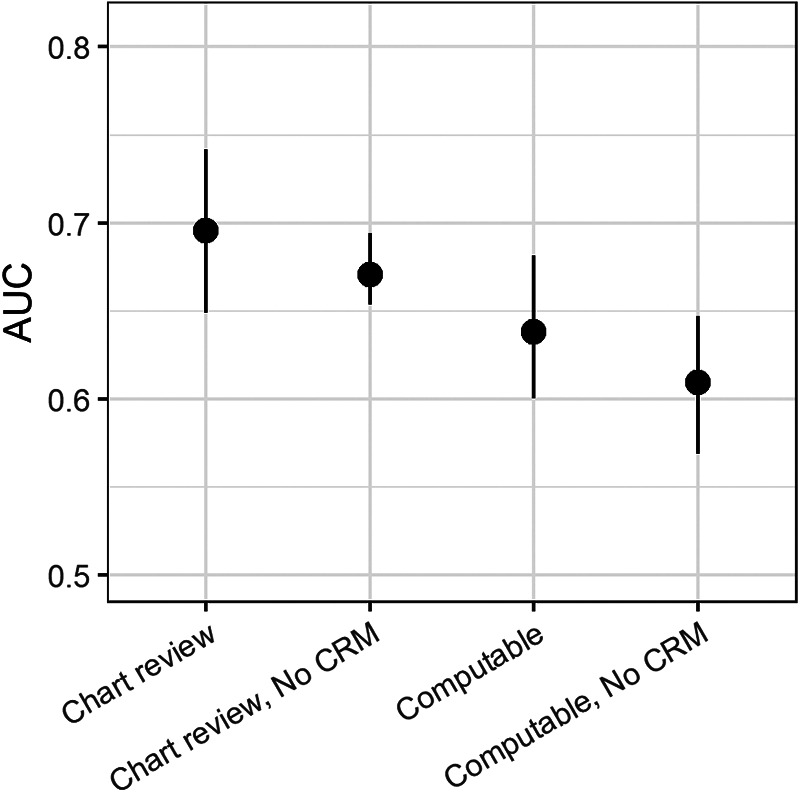
Area under the receiver operating characteristic (AUC) for predicting unplanned intubation based on chart review. For comparison, the AUC is also shown for combinations of models built to detect a computable phenotype and either using all features or excluding continuous monitoring data. Each AUC is based on 5-fold cross-validation with confidence intervals based on 200 bootstrap runs resampled by admission.

Figure 4 shows a sensitivity analysis for using a computable phenotype rather than chart review, and for excluding continuous cardiorespiratory monitoring features from the model. Each AUC is based on the cross-validated probabilities. Models using continuous cardiorespiratory monitoring features perform better than those without, and models based on chart reviewed events perform better than those based on a computable phenotype: the AUC using a computable phenotype is 29% lower than using manual chart review (0.638 and 0.696, respectively) and the AUC using chart review but excluding continuous cardiorespiratory monitoring features is 12% lower (0.671).

**Figure 5:**
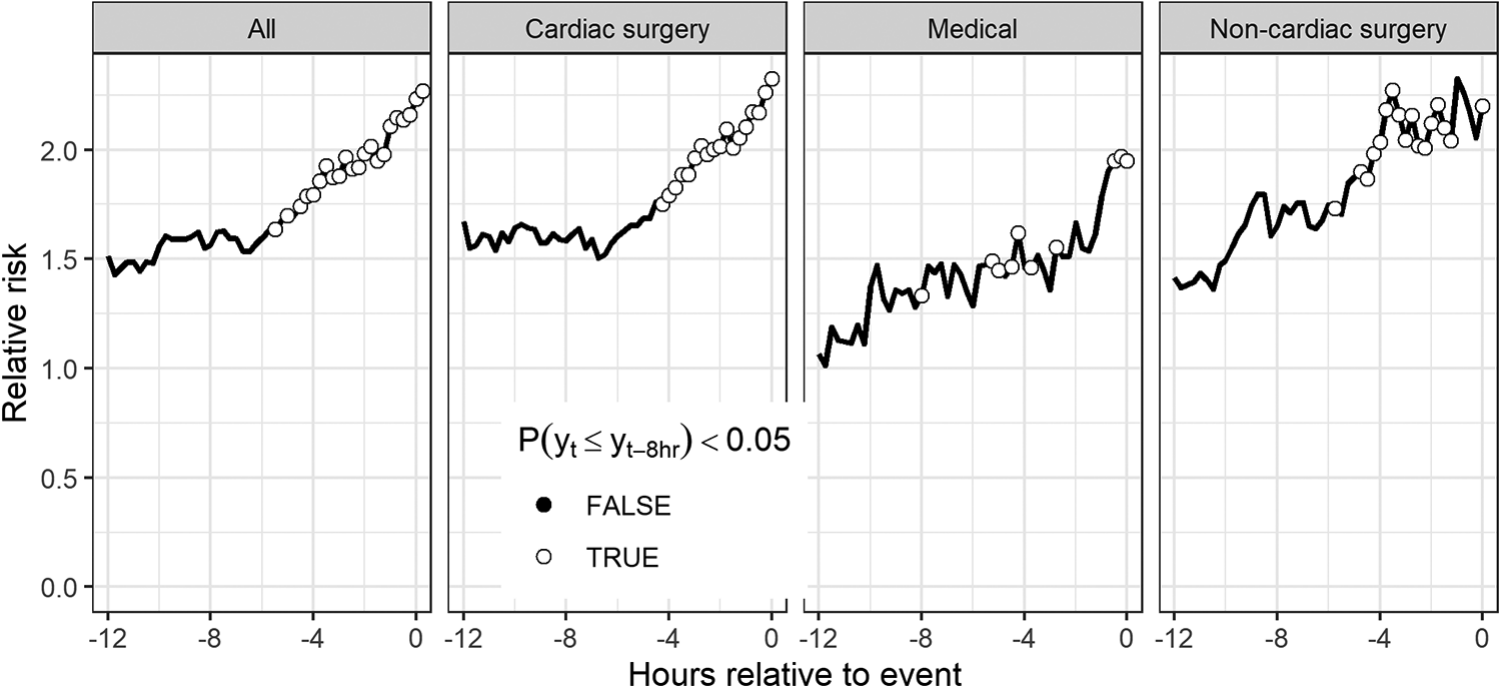
Average time series of model outputs leading up to the time of unplanned intubation. Results are cross-validated predictions from the model based on all patients and are shown for all patients as well as patients of each patient type. Open circles indicate risk estimates are significantly higher than risk estimates for the same patients 8 h prior based on a Wilcoxon signed rank test.

Figure 5 shows the average model output leading up to the time of unplanned intubation. For each event of unplanned intubation, we estimated the relative risks are based on the cross-validated predicted probabilities from the model. We then averaged the relative risks at each time to event for all events with data. To test that the predicted risk is increasing leading up to the time of unplanned intubation, we tested the null hypothesis that risk estimates are less than or equal to the values for the same patients 8 h prior. Open circles indicate that we reject the null hypothesis at the 0.05 significance level based on a Wilcoxon rank sum test. The figure indicates that all patient types have indications of physiological derangement 4–6 h prior to unplanned intubation.

## Discussion

Like adults, children have a physiologic signature of illness preceding unplanned intubation in the PICU. Generally, it comprises younger age, and abnormalities in electrolyte, hematologic and vital sign parameters. Given the heterogeneity of the patient population, it is not surprising that there are differences in the presentation among the major patient groups – medical, non-cardiac surgical, and cardiac surgical. We have argued elsewhere that predictive analytics monitoring is not a one-size-fits-all enterprise (30).

There are a variety of indications for unplanned intubation including oxygen failure, ventilation failure, and unstable hemodynamics. For the individual patient, it is often the case that multiple indications for intubation exist, demonstrating the complexity of physiology at play (31). For example, a patient with infantile botulism or spinal muscular atrophy may first exhibit signs of decompensation secondary to neuromuscular weakness which can progress to ventilation failure and ultimately oxygenation failure. Identifying physiologic and biochemical signatures of illness that help discriminate patients at risk for decompensation may allow for earlier intervention and potentially even prevention of the need for intubation.

We observed overlap in the signatures of illness between the medical and non-cardiac surgical patients. The leading indication for intubation in both the medical and non-cardiac surgical patients was respiratory failure (*e.g.*, oxygen and/or ventilation failure) typically driven by primary disorders of respiratory physiology (*e.g.*, pneumonia, acute lung injury). This is best illustrated by the heat map in Figure 2 exhibiting the influence of respiratory rate on the risk of intubation. Cardiac surgical patients, on the other hand, may be more likely to exhibit hemodynamic instability necessitating intubation, distinguishing them from the other two patient types.

This work is presented in the context that the multivariable statistical models that we use to look for signatures of illness can be repurposed for bedside risk prediction to supplement clinical decision making, including potential improvement in patient outcomes (32–36). The precedent is the heart rate characteristics index, a logistic regression model that used time series mathematical measures to identify early signatures of infection in preterm infants (37). Preterm infants in intensive care represent a mostly homogeneous cohort with a consistent signature of deterioration well-suited for logistic regression. The PICU, by contrast, may consist of multiple patient cohorts over a wide range of ages with different physiology and therefore physiological signatures, thus requiring more adaptable modeling approaches.

We found components of the signature in the labs (white blood cell count), vital signs (BP), and from the bedside monitor (every-2-second respiratory rate, variability of the SpO_2_). We affirmed the importance of continuous cardiorespiratory monitoring data in discovering dynamic signatures of illness (38–40) excluding it reduced the AUC by 12%. This finding underscores the importance of using all available sources of information in bedside predictive analytics monitoring (41).

Individual chart review proved to be of great importance in defining the signature of illness. When we used a computable phenotype, we found cases in 852 more patient admissions, but the statistical models returned poorer performance with a 29% reduction in AUC. Like sepsis, the gold standard for case identification for model training is individual chart review (42).

Our study has several limitations including the single-center observational nature. Our model was constructed using data from a mixed cardiac/medical-surgical PICU, limiting its applicability broadly to all PICUs and patient populations. We were limited to the time of the intubation, rather than the time the clinical decision to intubate was made, to define the event. We were also limited by the indication(s) for intubation (e.g., respiratory failure, hemodynamic instability) as recorded in the NEAR4KIDS database. To our knowledge, the inter-rater reliability for establishing intubation indication(s) for the NEAR4KIDS database has not been evaluated. The small number of admissions from teenagers, and the low rate of unplanned intubation in that age range, limits the ability to capture a complete signature of illness. Physiological monitoring data included epochs with artifact due to, for example, physical therapy or suctioning. We also did not control for the impact of routine medications, such as sedatives and vasoactives, or non-invasive respiratory support on physiology. We acknowledge that these factors contribute to the complex and nuanced clinical scenarios in the PICU, and that results of a predictive model are to be taken into consideration with many other factors.

Additionally, we were limited to the time of the intubation, rather than the time the clinical decision to intubate was made, to define the event. Several factors may influence the timing of the clinical decision to intubate. While one patient may demonstrate clinical decompensation over a period of hours and another may experience an acute catastrophic event, we were encouraged by the observation of improved AUC at four hours prior to the event. Next steps include external validation of our models and evaluation through prospective integration into clinical care.

We have studied physiological and biochemical dynamics leading up to the event of unplanned intubation in a PICU. We observed that patients who experienced unplanned intubation were younger, more likely to have a co-morbid condition, and more likely to have undergone cardiac surgery. In addition, we identified different signatures of illness for medical, cardiac surgical, and non-cardiac surgical patients. While all this information is already incorporated into clinical practice by PICU teams, using predictive analytics prospectively to accurately estimate risk based on high-dimensional physiological signatures can support clinical decision making, direct new approaches for continuous monitoring in the PICU, and improve patient outcomes.

## Data Availability

The datasets presented in this article are not readily available because the conditions of institutional approval do not provide for transmission of dataset outside of sponsoring institution. Requests to access the datasets should be directed to ms7uw@hscmail.mcc.virginia.edu.

## Supplementary material

**SUPPLEMENTARY FIGURE A1**

Histogram of time between ventilator respiratory rate entries in the electronic medical record on a logarithmic scale. Most entries are within 12 hours of the preceding entry.

**SUPPLEMENTARY FIGURE A2**

Empirical relative risk for cardiac surgery (left), medical (center), and non-cardiac surgery (right) patients as a function of age and each feature. On each panel, younger patients are to the left, and lower values of the feature are to the bottom. Each colored tile estimates the relative risk of unplanned intubation for each decile of age and the feature based on the surrounding quintile. Deciles remain the same across patient type panels. Higher relative risk is redder while lower is bluer.

**SUPPLEMENTARY FIGURE A3**

Marginal risk profiles for each feature in the random forest model. Note that marginal risk profiles average out any interdependence between features, such as the dependence of heart rate on age. The (natural) log odds of unplanned intubation in the next 12 hours (ordinate) is shown as a function of the value of each measured variable (abscissa) holding all other features at their median values.
