# Supplementary figures and images for: Signatures of illness in children requiring unplanned intubation in the pediatric intensive care unit: A retrospective cohort machine-learning study
Source: Front Pediatr. 2022 Oct 19;10:1016269. doi: 10.3389/fped.2022.1016269 (PMC9682496; doi:10.3389/fped.2022.1016269)

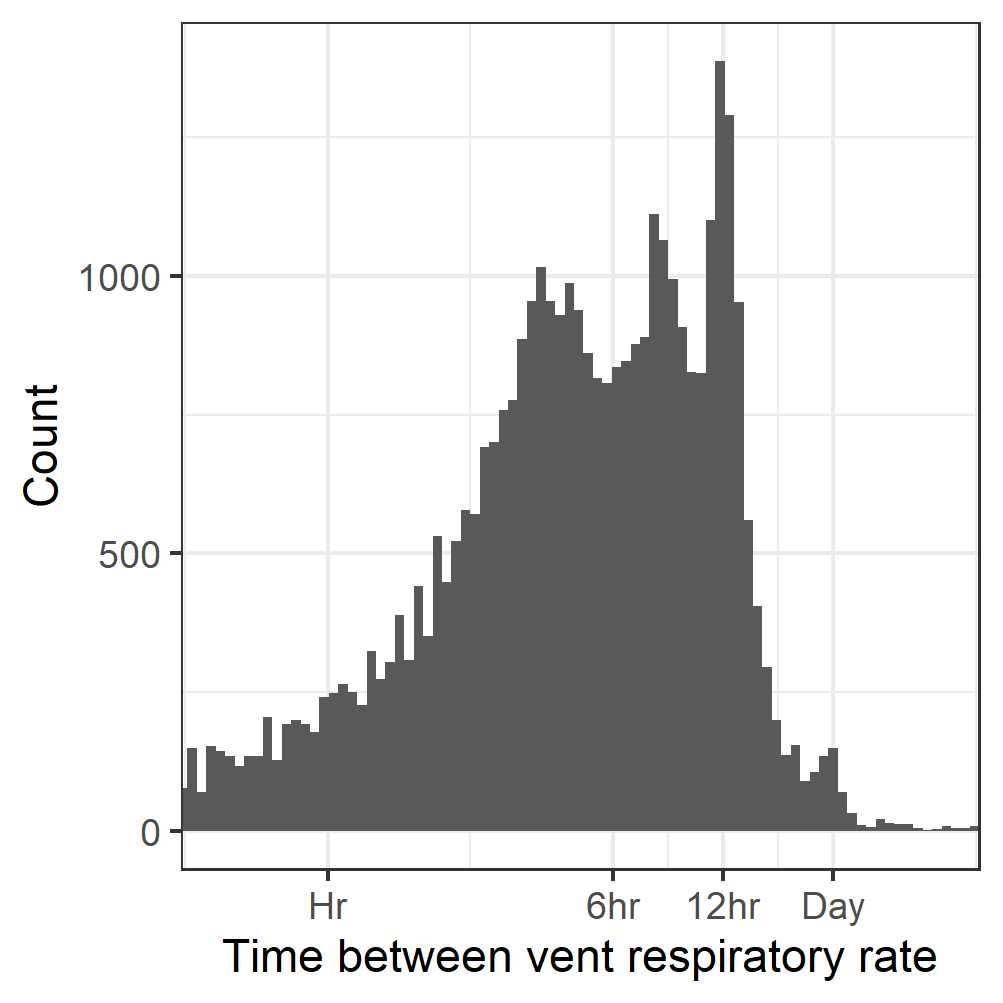

Supplement: Supplementary file 1 [file Image1.tiff]

(A)

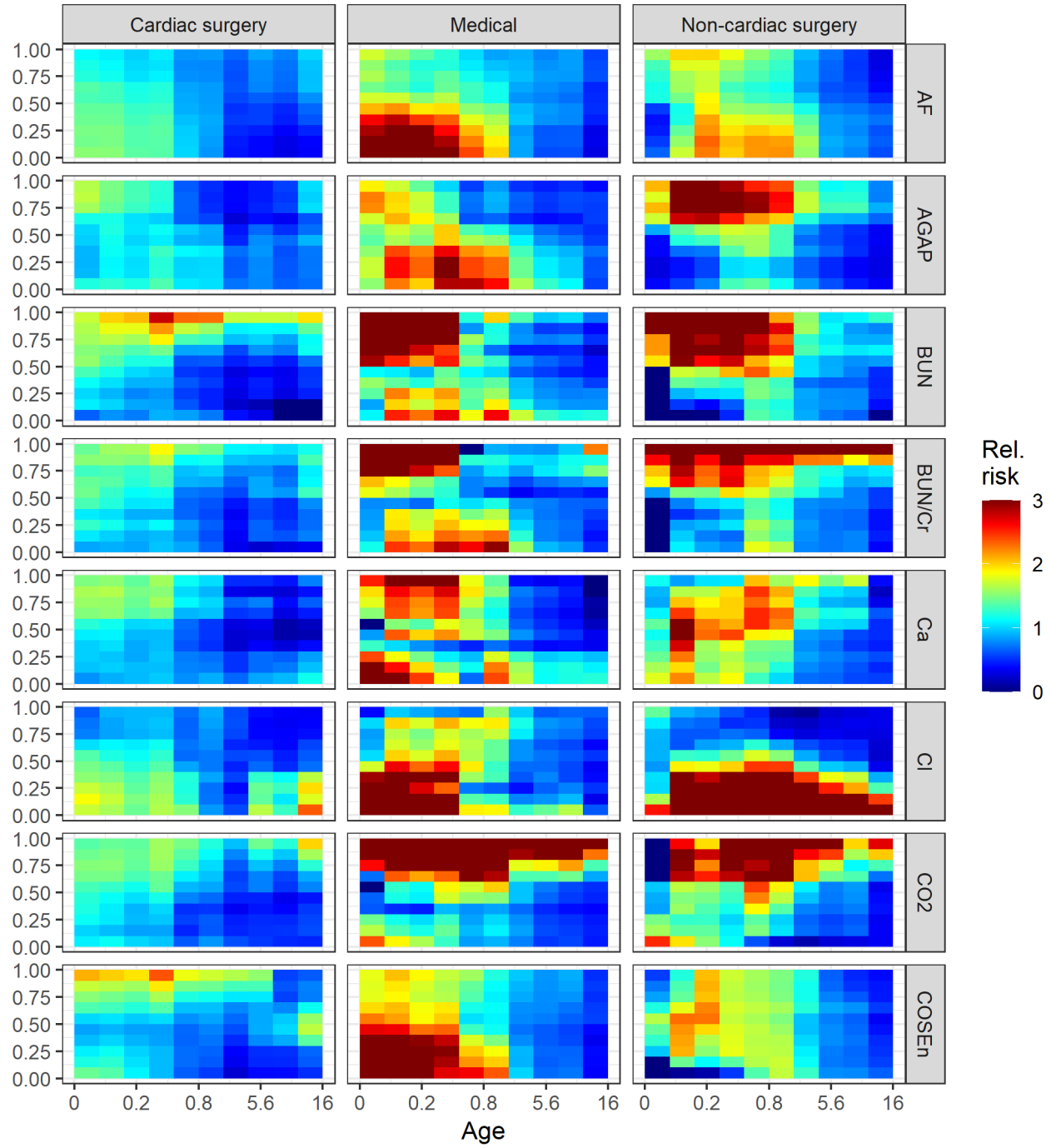

(B)

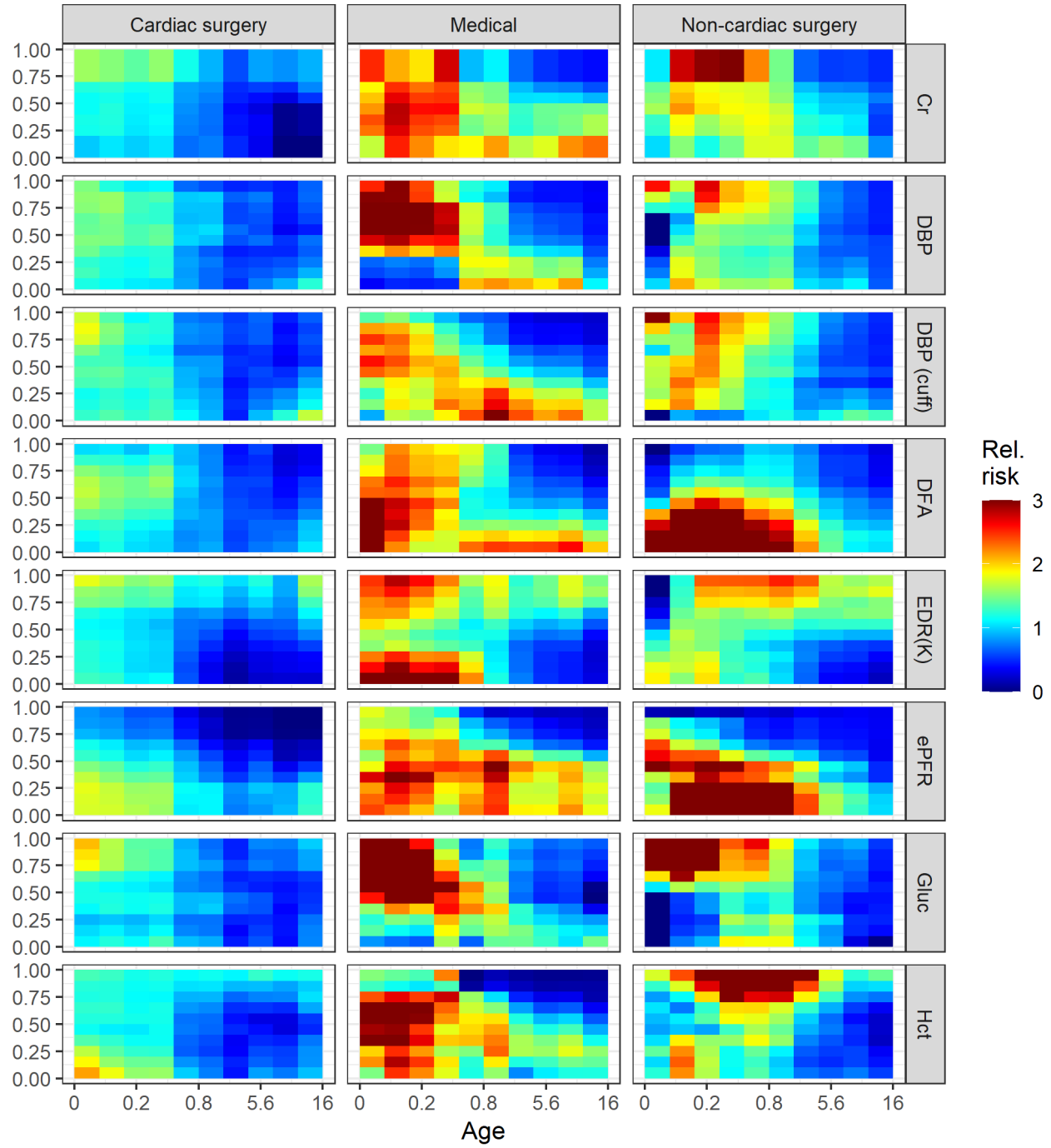

(C)

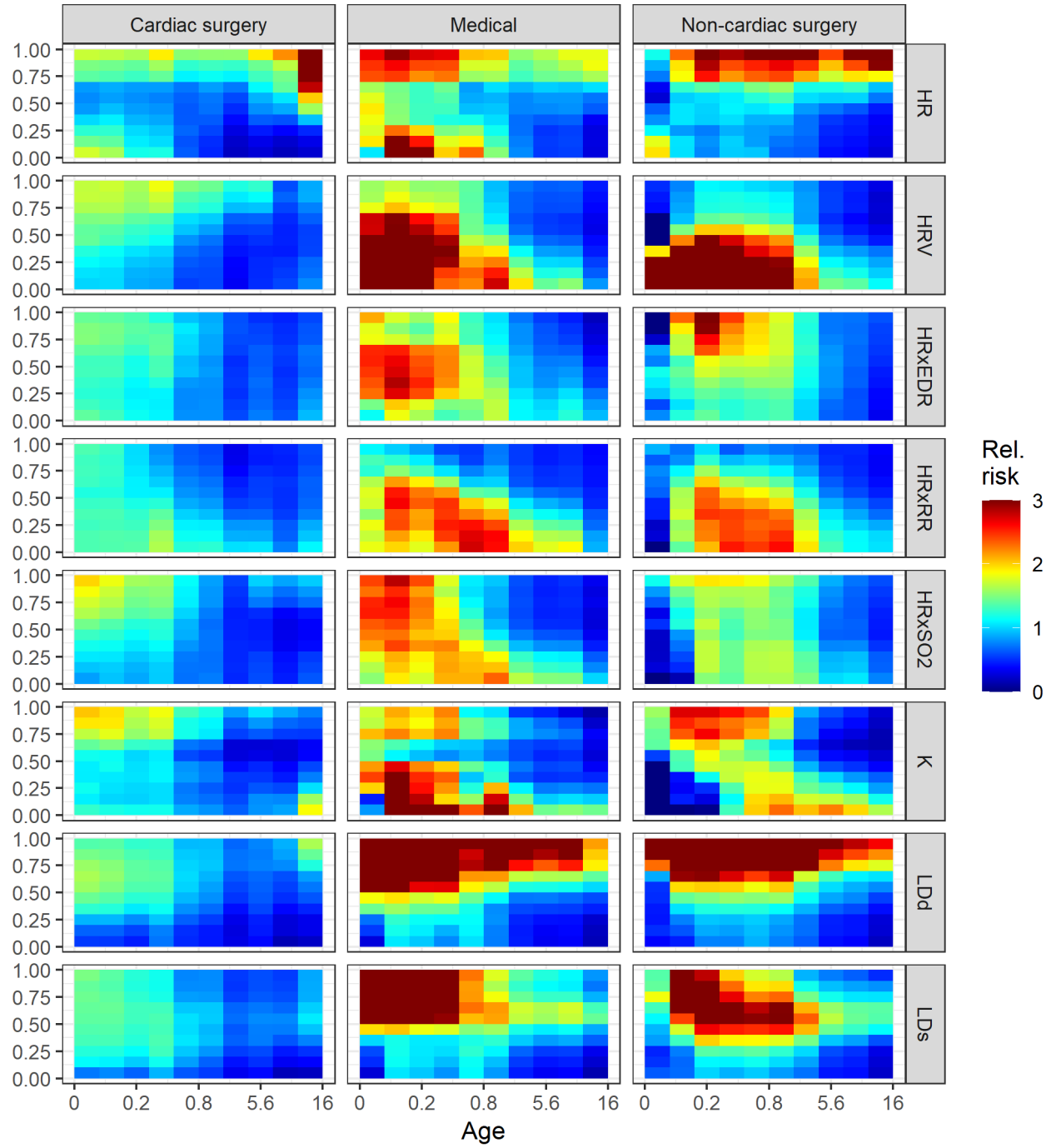

(D)

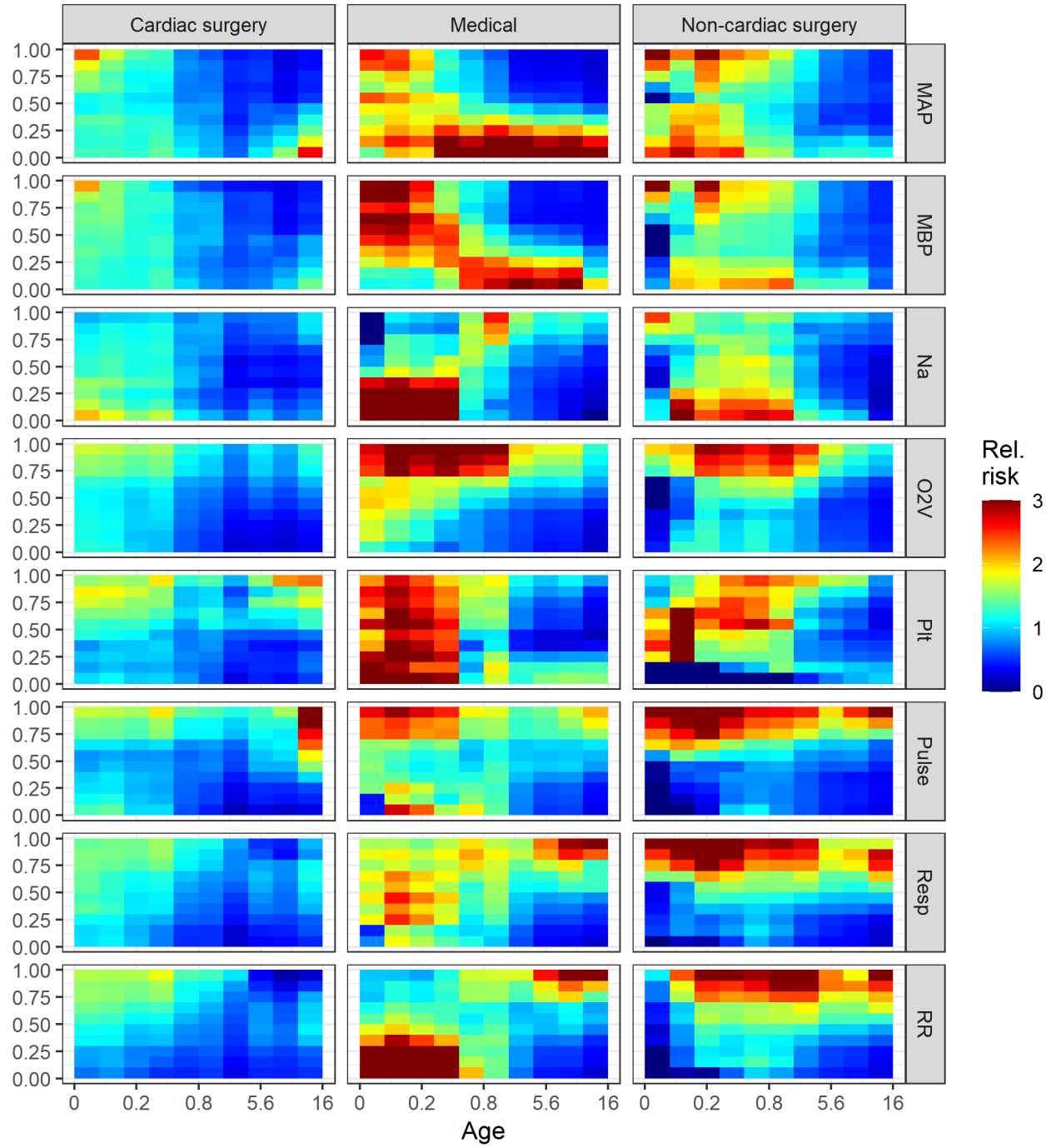

(E)

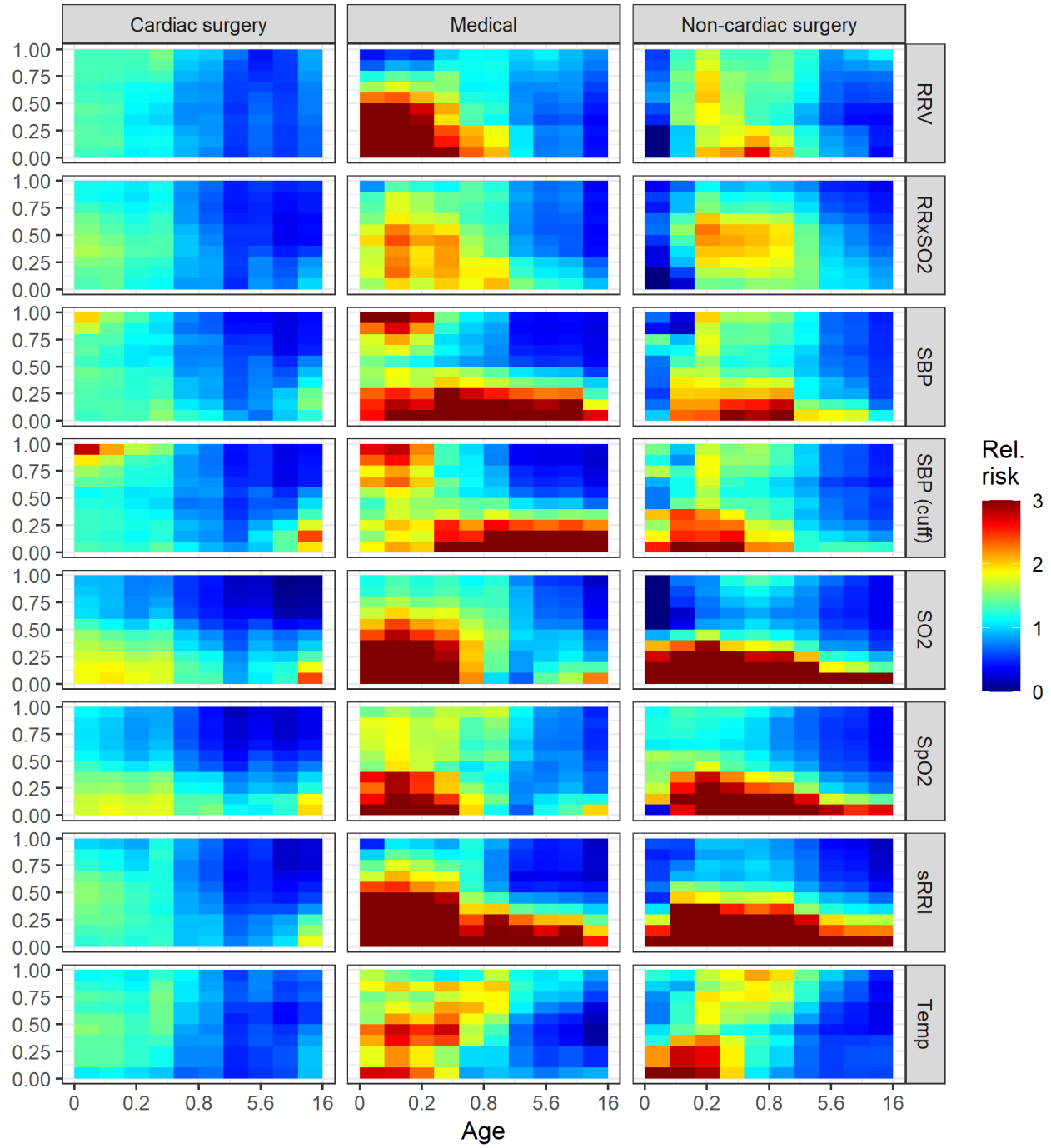

Supplement: Supplementary file 2 [file Image2.pdf]

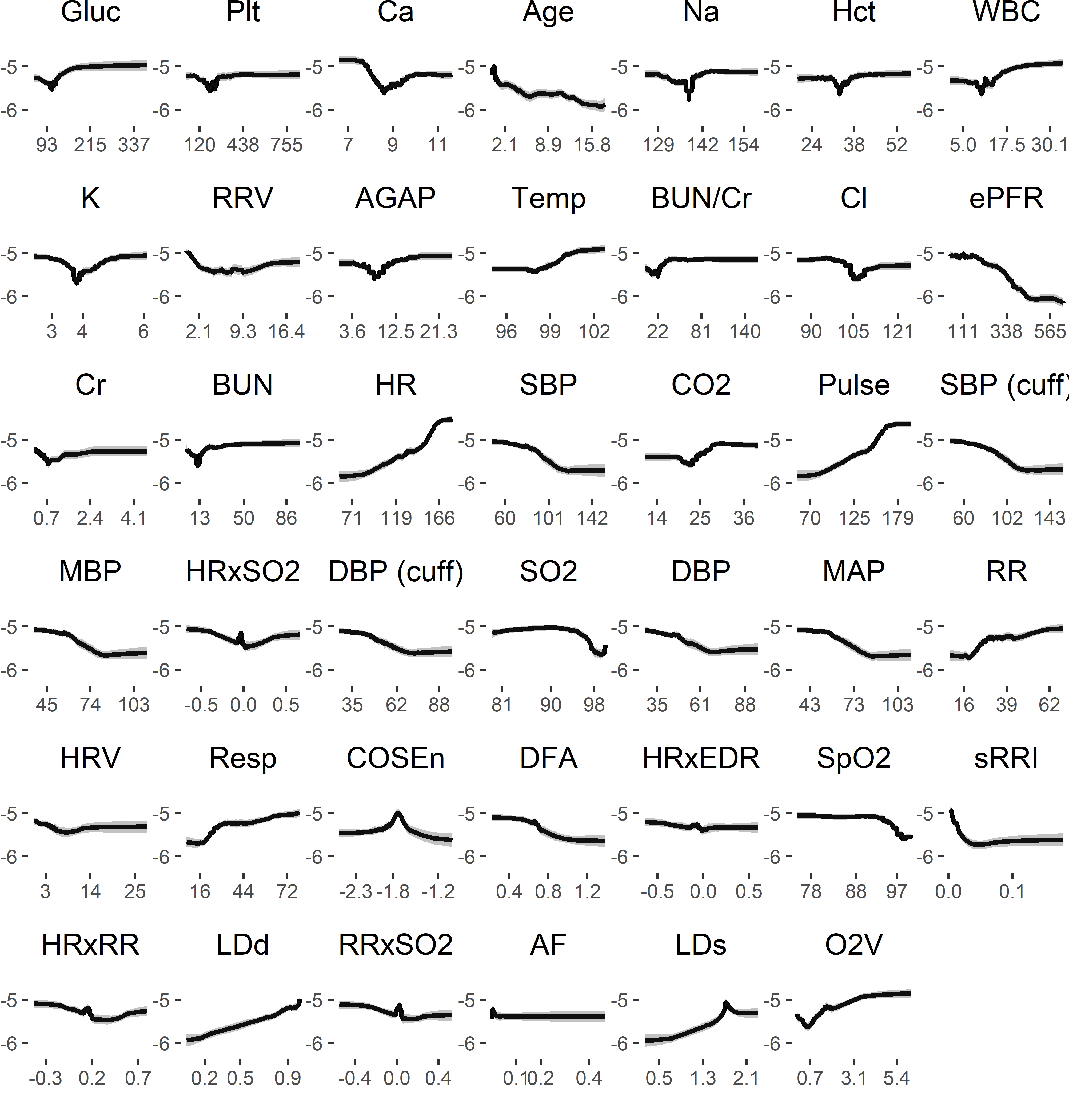

Supplement: Supplementary file 3 [file Image3.jpg]
